# *Gastrocopta* (Mollusca, Gastropoda, Pupillidae) in the Pilbara region of Western Australia

**DOI:** 10.3897/zookeys.261.4269

**Published:** 2013-01-24

**Authors:** Corey S. Whisson, Frank Köhler

**Affiliations:** 1 Western Australian Museum, Locked Bag 49, Welshpool, WA 6106; 2 Australian Museum, 6 College Street, Sydney, NSW 2010

**Keywords:** Australia, Pupilloidea, Pulmonata, 16S, COI

## Abstract

Six species of *Gastrocopta* have been identified from the Pilbara region, Western Australia, by means of comparative analyses of shell and mtDNA variation. Three of these species, *Gastrocopta hedleyi*, *Gastrocopta larapinta* and *Gastrocopta servilis*, have been recorded in the Pilbara for the first time. *Gastrocopta* sp. CW1 is probably new to science and might be endemic to the region. By contrast, *Gastrocopta hedleyi*, *Gastrocopta larapinta* and *Gastrocopta mussoni* are shown to be widespread.

## Introduction

*Gastrocopta* Wollaston, 1878 is the most speciose pupillid genus in Australia with twelve recorded species (Pokryszko 1996). Its members are found throughout most of Australia except for the humid south-west and south-east corners of the continent (Solem 1991; Pokryszko 1996; Stanisic et al. 2010). The Australian taxa have most recently been revised based on comparative shell morphology by Solem (1986, 1989) and Pokryszko (1996). Both works disagree on some details, mainly the morphological separation of *Gastrocopta larapinta* and *Gastrocopta mussoni* and the taxonomic distinctness within the size-variable *Gastrocopta margaretae* complex. Molecular studies that might help to resolve the taxonomic discrepancies have remained unavailable.

Previous works have focussed mainly on the northern, eastern and southern parts of coastal Australia and to a lesser degree on the mid-west and central parts of Australia (Pilsbry 1917; Iredale 1939; Solem 1986, 1989, 1991; Slack-Smith 1993; Pokryszko 1996; Köhler et al. 2012) while the fauna in Western Australian has remained poorly documented. In Western Australia most pupillid specimens have been collected along main roads of the more coastal areas and along major inland roads, but the interior of Western Australia has so far been widely neglected. Being of small size (maximum dimension less than 6mm) and cryptic in nature, pupillids are often ignored when documenting land snail diversity (Nekola 2009). The lack of specimens from inland areas of Western Australia has made it difficult to determine the relationships between west coastal specimens and those from central and eastern Australia.

Pilsbry (1917) in his world monograph on the subfamily Gastrocoptinae had little Australian material, except of a few types and vouchers received from Tate (from Central Australia) and Hedley (mostly from Eastern Australia). Solem (1986) was the first author to revise the Australian fauna more comprehensively. He examined the Pupillidae from the south and mid-west coasts of Western Australia and later (Solem 1989) the non-camaenid families (including Pupillidae) from the Kimberley, Northern Territory and Red Centre regions. A second major revision by Pokryszko (1996) extended the area of review in Western Australia only slightly, because only a small amount of additional material from the Western Australian Museum was studied (just 9 lots) and probably because the collection was little expanded since Solem (1986) examined the collections.

Since Pokryszko’s (1996) revision, the Western Australian Museum collection of *Gastrocopta* in the Pilbara region has greatly expanded. Most of this collecting has been associated with expanding mineral operations in the region and improved vehicle access to remote areas. A Western Australian Museum fieldtrip during August 2009 visited the eastern Pilbara area, collected macro- and micro- non-marine molluscs and significantly increased the pupillid collection in that region.

This paper (1) presents new data on *Gastrocopta* in the Pilbara, establishing new records and range extensions; (2) tests the taxonomic significance of morphological characters commonly used for the identification and delimitation of species by using a mitochondrial phylogeny; (3) provides comparative remarks on shell morphology of *Gastrocopta* species; (4) indicates systematic issues that require clarification by further studies. For detailed comparative analyses of shell characters we refer to Pokryszko (1996) and Solem (1986, 1989).

## Methods

All *Gastrocopta* material from the Pilbara in the malacological collections of the Western Australian Museum was examined. Additional specimens from the private collection of Mr Vince Kessner and from the collection of the Field Museum of Natural History, Chicago were also included. In total 545 *Gastrocopta* lots were studied with distributional maps being plotted by use of the online vector map software available at www.planiglobe.com.

Species identifications were based on shell characters, with particular emphasise on the size, shape and quantity of apertural barriers. Specimens were photographed and measured using a Leica MZ16A microscope with Leica DFC500 camera. DNA was extracted from entire specimens taken from their shell by use of a QIAGEN DNA extraction kit for animal tissue following the standard procedure of the manual. Fragments of the mitochondrial 16S rRNA (16S) and of the COI genes were amplified by PCR using the primer pairs: 16Sar and 16Sbr (Palumbi et al. 1991), and L1490 and H2198 (Folmer et al. 1994), respectively. Reactions were performed under standard conditions with an annealing step of 60 s at 55 °C for 16S and at 50 °C for COI. Both strands of purified PCR fragments were cycle sequenced by use of the PCR primers. Electropherograms were manually corrected for misreads, if necessary, and forward and reverse strands were merged into one sequence file using CodonCode Aligner v. 3.6.1 (CodonCode Corporation, Dedham, MA). Sequences have been deposited in GenBank (CO1: KC143966-KC143993, 16S: KC143994-KC144020). Sequence alignments were generated using MUSCLE as implemented in MEGA5 (Tamura et al. 2011). Uncorrected pair-wise genetic distances were calculated using MEGA5 under the option ‘pair-wise deletion of gaps’. Prior to the model-based phylogenetic analyses, the best-fit model of nucleotide substitution was identified for each gene fragment using the model proposal function of MEGA5. To infer phylogenetic relationships, we performed Maximum Likelihood (ML) analyses using MEGA5 with Nearest-Neighbor-Interchange (NNI) as heuristic method and automatic generation of the initial tree. Two-hundred ML bootstrap replicates were performed to assess the topology support.

**Figure 1.**
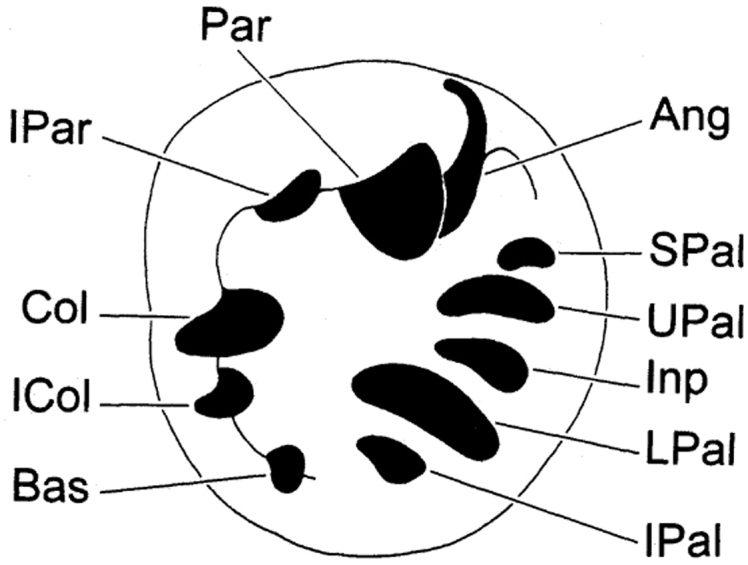
Apertural barriers of *Gastrocopta* (clockwise in aperture). **IPAR** Infraparietal Tooth **PAR** Parietoangular Tooth **ANG** Angular Tooth **SPAL** Suprapalatal Tooth **UPAL** Upper Palatal Tooth **INP** Interpalatal Tooth **LPAL** Lower Palatal Tooth **IPAL** Infrapalatal Tooth **BAS** Basal Tooth **COL** Columellar Tooth.

Abbreviations used for depositories of material are: FMNH, Field Museum of Natural History, Chicago, United States; VK, Vince Kessner Private Collection, Adelaide River, Australia; WAM, Western Australian Museum, Perth, Australia. For shell aperture barrier terminology we followed Pokryszko (1996), reproduced here in Fig. 1.

## Taxonomic part

Six species of *Gastrocopta* were recorded from the Pilbara region (Table 1). Four species are endemic to Australia, one species is introduced and one species requires further investigation (*Gastrocopta* sp. CW1). Another species, *Gastrocopta bannertonensis* was only collected from the inner mid-west region of Western Australia and was not discussed in this paper.

**Table 1.** Mean maximum shell height and width of *Gastrocopta* species from the Pilbara region.

**Species**	**N**	**Mean max. shell height (mm)**	**Mean max. shell width (mm)**
*Gastrocopta larapinta*	17	2.273	1.115
*Gastrocopta larapinta* (Kalgan Pool)	3	2.311	1.042
*Gastrocopta mussoni* (ovate)	7	2.278	1.251
*Gastrocopta mussoni* (cylindrical)	7	2.212	1.064
*Gastrocopta hedleyi*	14	2.017	1.014
*Gastrocopta margaretae* (Pilbara)	14	2.128	0.955
*Gastrocopta margaretae* (SWA)	13	2.466	1.128
*Gastrocopta* sp. CW1	14	1.664	0.895
*Gastrocopta servilis*	14	2.255	1.070

### 1. Gastrocopta hedleyi

Pilsbry, 1917

http://species-id.net/wiki/Gastrocopta_hedleyi

Fig. 2B

Gastrocopta hedleyi Pilsbry 1917 [in 1916-1918]: 166-167, pl. 27, figs 1–4; Solem 1991: 250; Pokryszko 1996: 1104, fig. 18; Stanisic 1998: fig. 17.42e; Stanisic et al. 2010: 102–103.
Australbinula hedleyi Iredale 1937a: 301.

#### Type locality.

Narrabri, New South Wales.

#### Material studied.

Western Australia: Abydos (64km W of Marblebar): 21.1343°S, 119.1259°E (WAM S64439). Burrup Peninsula: 20.6080°S, 116.7670°E (WAM S60089); 20.6141°S, 116.7548°E (WAM S60226); 20.6066°S, 116.7681°E (WAM S60227); 20.5833°S, 116.8000°E (WAM S60228, WAM S60230, WAM S60349, WAM S61117-21); 20.6102°S, 116.7607°E (WAM S60353); 20.6232°S, 116.7784°E (WAM S60402); 20.6166°S, 116.7833°E (WAM S60475); 20.6119°S, 116.7587°E (WAM S60477); 20.6238°S, 116.7777°E (WAM S60480); 20.6300°S, 116.7800°E (WAM S65167); 20.5858°S, 116.8044°E (WAM S65168). Cloud Break area: 22.2997°S, 119.3737°E (WAM S60416). Hope Downs: 23.0865°S, 119.3184°E (WAM S42661); 23.0379°S, 119.2124°E (WAM S42663); 23.0952°S, 119.2022°E (WAM S59553); 23.1030°S, 119.2917°E (WAM S59555). Kalgan Pool area: 23.1872°S, 119.6958°E (WAM S58079); 23.1877°S, 119.6965°E (WAM S58091). Kangeenarina Gorge: 22.0588°S, 117.8549°E (WAM S60085). Karajini National Park: 22.4782°S, 118.5598°E (WAM S65307); 22.9797°S, 118.5891°E (WAM S65310); 22.8446°S, 118.5403°E (WAM S65314); 22.3714°S, 118.2989°E (WAM S65336). Marillana Station: 22.4285°S, 119.2043°E (WAM S81440). Mount Brockman area: 22.4815°S, 117.2384°E (WAM S83560). Mt Farquhar area: 22.4815°S, 116.8108°E (WAM S83564); 22.4932°S, 116.8679°E (WAM S83586). Nullagine area: 22.3848°S, 119.9696°E (WAM S58093); 22.3210°S, 119.4442°E (WAM S80958). Orebody 35°E(ca. 8km W of Newman): 23.4047°S, 119.6052°E (WAM S64713); 23.3943°S, 119.6316°E (WAM S64715-6, WAM S64718, WAM S64734, WAM S64753); 23.4108°S, 119.5715°E (WAM S64720); 23.3994°S, 119.5843°E (WAM S64722); 23.4045°S, 119.6211°E (WAM S64726); 23.4182°S, 119.5847°E (WAM S64730, WAM S64744); 23.4049°S, 119.6052°E (WAM S64732); 23.4045°S, 119.6247°E (WAM S64735); 23.4049°S, 119.6053°E (WAM S64737, WAM S64747); 23.4003°S, 119.6524°E (WAM S64740); 23.4137°S, 119.5826°E (WAM S64741); 23.4029°S, 119.6021°E (WAM S64742); 23.4003°S, 119.5721°E (WAM S64745); 23.3947°S, 119.5913°E (WAM S64750). ca. 35km E of Paraburdoo: 23.1300°S, 117.8984°E (WAM S41446); 23.1663°S, 117.9484°E (WAM S41447); 23.1670°S, 117.9597°E (WAM S41448). ca. 7.07.5km NW of Tom Price: 22.6500°S, 117.7185°E (WAM S42668); 22.6423°S, 117.7451°E (WAM S42672). Phil’s Creek: 22.7319°S, 119.1940°E (WAM S59376). Sulphur Springs: 21.1475°S, 119.2269°E (WAM S60229). Wonmunna: 23.1216°S, 119.0498°E (WAM S65971); 23.1428°S, 119.0099°E (WAM S65976); 23.1266°S, 119.0470°E (WAM S65992, WAM S81027, WAM S81085); 23.1255°S, 119.0797°E (WAM S80937); 23.1287°S, 119.0904°E (WAM S80938); 23.1393°S, 119.0182°E (WAM S80939); 23.1220°S, 119.0611°E (WAM S80941); 23.1436°S, 119.0064°E (WAM S81001, WAM S81073); 23.1185°S, 119.0649°E (WAM S81004); 23.1592°S, 118.9928°E (WAM S81025, WAM S81062); 23.1632°S, 118.9770°E (WAM S81033, WAM S81054, WAM S81168, WAM S81176); 23.1615°S, 119.0020°E (WAM S81048, WAM S811056); 23.1185°S, 119.0649°E (WAM S81091); 23.1596°S, 118.9703°E (WAM S81052); 23.1283°S, 119.0736°E (WAM S81059); 23.1331°S, 119.0154°E (WAM S81103); 23.1356°S, 119.0463°E (WAM S81120); 23.1546°S, 118.9932°E (WAM S81122); 23.1314°S, 119.0774°E (WAM S81174). ca. 6km W of Wodgina Mine: 21.2383°S, 118.6519°E (WAM S65895); 23.1348°S, 119.0338°E (WAM S81074).

#### Distribution.

This species has previously been recorded fromnorthern New South Wales and from scattered localities in northern Queensland (Cape York Peninsula), central Australia (Glen Helen area) and northern Western Australia (King Leopold Ranges) (Pokryszko 1996). In addition, it is now recorded from the Hamersley Ranges, the Burrup Peninsula and a few isolated sites from approximately 100 km SSE of Port Hedland in the Pilbara region (Figure 3).

#### Comparative morphology.

*Gastrocopta hedleyi* shellsare slightly smaller (shorter) than those of other *Gastrocopta* species (excluding *Gastrocopta* sp. CW1) recorded from the Pilbara. They typically have (1) a large, usually rounded (sometimes acute) columellar tooth that is drooping at the anterior end (2) a high, strongly convergent upper palatal tooth (3) a long, high, strongly twisted parietoangular tooth that usually comes in close proximity to the upper palatal tooth (4) a prominent infraparietal tooth that is sometimes prolonged as thin ridge on parietal wall (5) often a strong basal tooth (6) very occasionally with a weak interpalatal tooth.

Some *Gastrocopta hedleyi* shells (particularly more elongate specimens) can be difficult to separate from the ovate form of *Gastrocopta mussoni* but (1) are smaller (slender) when sympatric (2) have a less rounded body whorl (3) have a more strongly sigmoid lower palatal tooth (4) have a larger upper palatal tooth that is usually strongly convergent with the lower palatal (5) have a longer, more strongly twisted parietoangular tooth (6) have a larger, more rounded columellar tooth that is usually drooping at the anterior end .

The cylindrical form of *Gastrocopta mussoni* is also very similar to *Gastrocopta hedleyi* but (1) has a lower, shorter and less twisted parietoangular tooth (usually at 45^o^ angle in apertural view) (2) has a shorter and less sigmoid (usually straight) lower palatal tooth (3) generally lacks an infraparietal tooth (4) has a smaller upper palatal tooth (occasionally slightly convergent with lower palatal) (5) has a more acutely angled, slanted columellar tooth, rarely drooping at the anterior end.

#### Remarks.

There is considerable variation in the shell size and barrier length of specimens identified as *Gastrocopta hedleyi* during this study. Many specimens grouped as *Gastrocopta hedleyi* from the eastern Hamersley Range (eg. Wonmunna, Kalgan Pool) have reduced barriers and often a lower parietoangular tooth (nearing 45^o^ angle in apertural view) but a large series shows a progression to shells that typically possess a large, strongly convergent upper palatal tooth and a strongly twisted parietoangular tooth.

Solem (1989) mentioned that *Gastrocopta hedleyi* was somewhat similar to *Gastrocopta pilbarana*, although in that case he was actually referring to the ovate form of *Gastrocopta mussoni* (see section on *Gastrocopta mussoni*).

The abundance and seemingly allopatric nature of *Gastrocopta hedleyi* on the Burrup Peninsula is intriguing. The large numbers are presumably related to its’ habitat requirement of either Fig tree, Cypress or Brigalow Stands among rocky substrates, and its’ preference for high calcium soils (Pokryszko 1996). Away from the Burrup Peninsula, the distribution of *Gastrocopta hedleyi* is somewhat patchy and is probably related to the isolated occurrence of its’ preferred vegetative structures among rocks as well as less alkaline soils.

**Figure 2.**
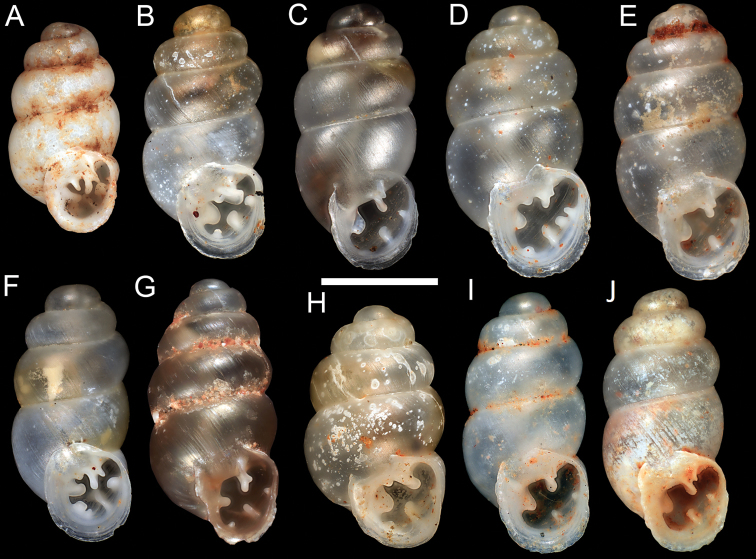
*Gastrocopta* species: **A** *Gastrocopta* sp. CW1 (WAM S60408, Exmouth) **B** *Gastrocopta hedleyi* Pilsbry, 1917 (WAM S61117, Burrup Peninsula) **C–E** *Gastrocopta larapinta* (Tate, 1896) **C** WAM S58005, Kalgan Pool **D–E** WAM S60368, Roy Hill Station **F** *Gastrocopta margaretae* (Cox, 1868) (WAM S42834, Bateman Sanctuary); G *Gastrocopta servilis* (Gould, 1843) (WAM S60237, Karratha) **H–J** *Gastrocopta mussoni* Pilsbry, 1917 **H** Ovate Form (WAM S42865, Roy Hill Station) **I** Cylindrical Form (WAM S59375, Phils` Creek) **J** Cylindrical Form (WAM S61070), Roy Hill Station) . (Scale Bar = 1mm).

**Figure 3.**
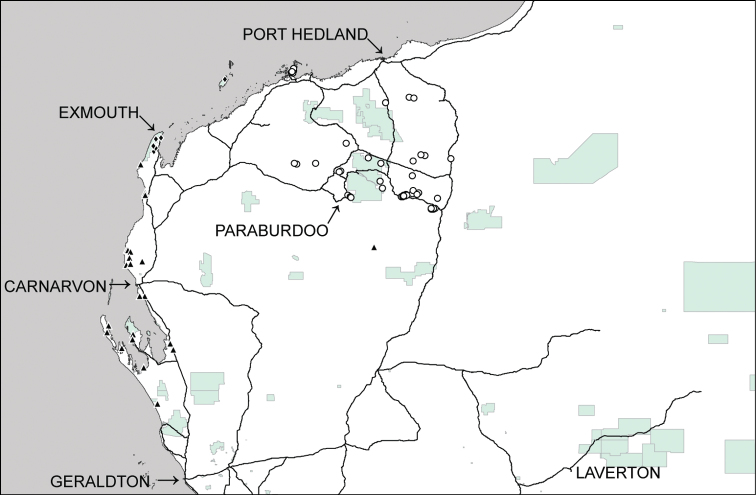
Distributional records of *Gastrocopta hedleyi* Pilsbry, 1917 (○), *Gastrocopta margaretae* (Cox, 1868) (▲) and *Gastrocopta* sp. CW1 (♦) in the Pilbara region. Shaded: Protected area.

### 2. Gastrocopta larapinta

(Tate, 1896)

http://species-id.net/wiki/Gastrocopta_larapinta

Fig. 2C–E

Pupa larapinta Tate 1896: 205–206, pl. 19, figs 19a–b.
Gastrocopta larapinta Pilsbry 1917 [in 1916–1918]: 168–171, pl. 30, figs 5–7,9–11; Solem 1989; 490–491, figs 54–55; 1991: 249; Pokryszko 1996: 1109–1110, figs 19, 22.
Australbinula larapinta Iredale 1937a: 302; 1937b: 10.

#### Type locality.

Central Australia.

#### Material studied.

Western Australia: Cane River: 22.0298°S, 115.4296°E (WAM S42993). Central Pilbara: 22.3855°S, 117.4667°E (WAM S58492, WAM S65790); 22.3200°S, 117.6194°E (WAM S65778); 22.2675°S, 117.7197°E (WAM S65780); 22.1350°S, 117.4728°E (WAM S58493); 21.0216°S, 117.0560°E (WAM S65817); 20.7506°S, 117.0096°E (WAM S65797). Christmas Creek: 22.4061°S, 119.7376°E (WAM S65612); 22.3985°S, 119.7930°E (WAM S65604). Cloud Break area: 22.3210°S, 119.4442°E (WAM S34453, WAM S80956); 22.2688°S, 119.3147°E (WAM S61128); 22.3652°S, 119.3409°E (WAM S65150); 22.3985°S, 119.4748°E (WAM S65156); 22.3949°S, 119.5019°E (WAM 65161); 22.3527°S, 119.4189°E (WAM S65135). Collier Rocks: 20.4071°S, 116.8514°E (VK 30297). Cy Creek: 22.8183°S, 114.0609°E (WAM S34381). Du Boulay Creek: 21.1833°S, 116.1833°E (WAM S34569). Fortescue Marsh area: 22.2930°S, 119.0606°E (WAM S61996-7); 22.4646°S, 119.7726°E (WAM S64651, WAM S64654); 22.4430°S, 119.7785°E (WAM S64649); 22.4252°S, 119.7235°E (WAM S64635); 22.3125°S, 119.2348°E (WAM S64634, WAM S64637); 22.2875°S, 119.1701°E (WAM S64650); 22.2822°S, 119.1276°E (WAM S42923, WAM S64644, WAM S64652, WAM S64694). Hope Downs: 23.1030°S, 119.5821°E (WAM S59298); 23.0919°S, 119.1875°E (WAM S59550). Jimblebar: 23.3693°S, 120.1958°E (WAM S41346). Kalgan Pool area: 23.1877°S, 119.6965°E (WAM S58005); 23.1874°S, 119.6957°E (WAM S80939). Koodaideri Corridor West (90.4km NW of Tom Price): 21.8833°S, 117.7000°E (WAM S83434). Marillana Station: 22.6260°S, 119.2834°E (WAM S34638); 22.5840°S, 119.3114°E (WAM S34632, WAM S34472); 22.5739°S, 119.2562°E (WAM S34474); 22.5666°S, 119.2327°E (WAM S34637); 22.5663°S, 119.2308°E (WAM S34633); 22.6013°S, 119.2913°E (WAM S80924); 22.5625°S, 119.2311°E (WAM S80908); 22.1269°S, 119.2123°E (WAM S80911); 22.4080°S, 119.0067°E (WAM S80914); 22.4285°S, 119.2043°E (WAM S8143940); 22.3476°S, 119.1518°E (WAM S81433); 22.4295°S, 119.1923°E (WAM S81446). Millstream National Park: 21.2000°S, 117.2667°E (WAM S60343); 21.6000°S, 117.1000°E (WAM S 61044); 21.5833°S, 117.1000°E (WAM S 61048); 21.5833°S, 117.0833°E (WAM S60944-5); 21.5833°S, 117.0667°E (WAM S 60947); 21.4255°S, 117.0535°E (WAM S81213); 21.2039°S, 117.0440°E (WAM S81267). ca. 30km NNE of Newman: 23.1164°S, 119.8865°E (WAM S64469). ca. 65km NW of Newman: 22.9169°S, 119.2128°E (WAM S80937). ca. 108118km N of Newman: 22.3132°S, 119.8599°E (WAM S65534, WAM S65673); 22.3134°S, 119.7886°E (WAM S65646); 22.2972°S, 119.8633°E (WAM S65530, WAM S65682); 22.2954°S, 119.8109°E (WAM S65652). North Star Mine: 21.2284°S, 119.0386°E (WAM S65720); 21.2104°S, 118.8769°E (WAM S65723). Phil’s Creek: 22.7412°S, 119.1959°E (WAM S59388, WAM S80934); 22.7320°S, 119.1836°E (WAM S59374). ca. 100km S of Port Hedland: 20.6066°S, 119.5016°E (WAM S80942). ca. 200km SSE of Port Hedland: 22.1554°S, 119.0216°E (WAM S83486). Robe River area: 21.8063°S, 116.0774°E (WAM S42832). 6km SW of Redmont Airport: 22.0195°S, 118.9816°E (WAM S83423). NNE of Rocklea Homestead: 22.7882°S, 117.4974°E (WAM S80977). Roy Hill Station: 22.4898°S, 119.8951°E (WAM S34703); 22.4547°S, 119.8709°E (WAM S42924, WAM S60428-9, WAM S65389); 22.4943°S, 119.9217°E (WAM S60359, WAM S60368, WAM S60373, WAM S60378); 22.5383°S, 119.9424°E (WAM S42831, WAM S42833, WAM S60232, WAM S60857, WAM S60861); 22.4793°S, 119.9420°E (WAM S42925, WAM S60363, WAM S60426, WAM S60853, WAM S60860); 22.6394°S, 119.9642°E (WAM S60398); 22.5769°S, 119.9952°E (WAM S603667, WAM S608545, WAM S60859); 22.5771°S, 120.0247°E (WAM S60422); 22.7058°S, 119.7082°E (WAM S60392); 22.6430°S, 119.9599°E (WAM S60396); 22.6076°S, 119.9826°E (WAM S60388); 22.6593°S, 119.9198°E (WAM S60397); 22.6642°S, 119.9458°E (WAM S61066, WAM S61069); 22.6431°S, 119.9642°E (WAM S61075, WAM S61077); 22.6225°S, 119.9634°E (WAM S610723); 22.5050°S, 119.9143°E (WAM S64455). Running Waters (east of Nullagine): 21.6819°S, 121.1254°E (WAM S58039, WAM S58050); 21.6806°S, 121.1261°E (WAM S58032). 15km W of Shaw River Airport: 21.6123°S, 119.2642°E (WAM S83412). Wonmunna: 23.1220°S, 119.0611°E (WAM S81087, WAM S81182); 23.1356°S, 119.0463°E (WAM S81107, WAM S81125, WAM S81164); 23.1216°S, 119.0498°E (WAM S81096); 23.1201°S, 119.0484°E (WAM S65993, WAM S80904). ca. 18-23km SE of Wodgina Mine: 21.2273°S, 118.8336°E (WAM S646089); 21.2871°S, 118.8671°E (WAM S64604, WAM S64616). ca. 20km NNE of Wodgina Mine: 21.0260°S, 118.7024°E (WAM S64610). Yule River area: 21.6961°S, 118.8604°E (WAM S83372).

#### Distribution.

**Figure 4.**
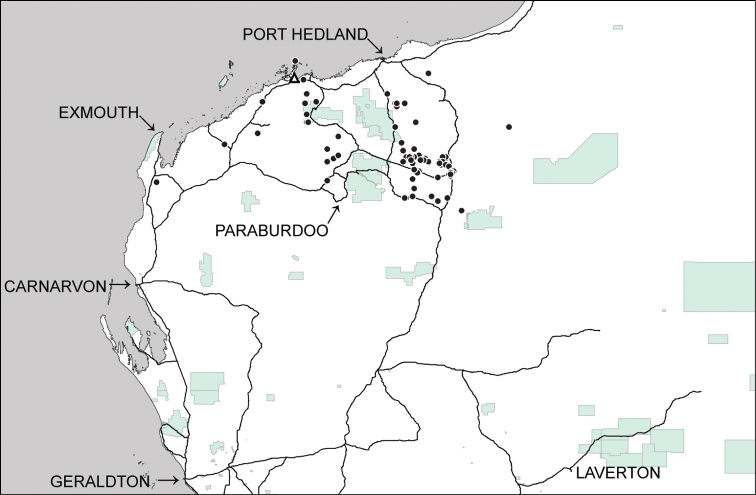
Distributional records of *Gastrocopta larapinta* (Tate, 1896) (●) and *Gastrocopta servilis* (Gould, 1843) (Δ) in the Pilbara region. Shaded: Protected area.

This species has previously been recorded fromcentral Australia (southern part of Northern Territory) with fewer records in north-western Queensland (Gregory River Basin); eastern coast of Queensland and a single record from the Oscar Ranges, in the southern Kimberley region of Western Australia (Pokryszko 1996). In addition, it is now recorded from throughout most of the Pilbara region, but is surprisingly absent from near coastal areas and islands (Figure 4).

#### Comparative morphology.

The shells of typical *Gastrocopta larapinta* specimens are distinguished from most other *Gastrocopta* species in the Pilbara by (1) their large size (2) usually the presence of three solid palatal teeth (the interpalatal tooth varying from a tiny callus to large tooth) (3) a short, solid parietoangular tooth that is usually deflected or curved moderately toward the columellar wall so that its anterior end is somewhat vertical in the apertural view (4) a long angular tooth that is generally fused (or connected via a translucent callus) to the parietoangular tooth, occasionally separate (particularly those that lack or have a small interpalatal tooth) (5) smaller, more rounded columellar tooth that curves or angles abruptly toward the columellar wall (6) usually the presence of an infraparietal tooth or basal tooth (or both).

Typical *Gastrocopta larapinta* shells with a small interpalatal tooth (or very occasionally no interpalatal tooth) appear considerably more variable in apertural barrier structure (particularly in high calcareous soils), making their separation from the cylindrical and elongate-ovate forms of *Gastrocopta mussoni* difficult. As such the following separation is tentative. *Gastrocopta larapinta* shells are typically (1) slightly to moderately larger (obese) (2) have a slightly smaller, more rounded columellar tooth (3) usually a much shorter parietoangular tooth that is positioned lower in apertural view (4) generally possesses an infraparietal tooth (5) often a slightly lower, less convergent upper palatal tooth (6) usually a less sigmoid lower palatal tooth.

#### Remarks.

The separation of *Gastrocopta larapinta* (small or no interpalatal tooth) with the cylindrical and elongate-ovate forms of *Gastrocopta mussoni* has proved extremely difficult and a more detailed molecular study is required to resolve this issue. Pokryszko (1996) separated these *Gastrocopta larapinta* specimens from cylindrical *Gastrocopta mussoni* based on shell size (smaller) and columellar tooth angle (less acute). However, from the small genetic data available and from examination of many shells, *Gastrocopta larapinta* shells were slightly, to moderately more obese.

Some of those near west coast specimens (Cy Creek) included as cylindrical *Gastrocopta mussoni* contained mixed lots of *Gastrocopta larapinta* and *Gastrocopta mussoni*. Interestingly, Poykryszko had identified a few of these larger Cy Creek specimens as *Gastrocopta larapinta*, but included those records as *Gastrocopta mussoni* in her publication. Pokryszko (1996) also noted in a large lot of *Gastrocopta mussoni* from central Australia (FMNH 201570) that some of the ovate *Gastrocopta mussoni* were quite large (many having an interpalatal tooth) but we consider most of those to be *Gastrocopta larapinta* with a small or no interpalatal tooth *(*see *Gastrocopta mussoni* section*)*.

It is possible Pokryszko (1996) was alluding to a slender form of *Gastrocopta larapinta* when separating cylindrical *Gastrocopta mussoni* and *Gastrocopta larapinta* (no interpalatal tooth) but this does not reflect accurately in her identifications. During this study specimens from Kalgan Pool (WAM S58005) were unexpectedly grouped within the *Gastrocopta larapinta* clade. These specimens, although slightly more slender, have proven difficult to separate from cylindrical *Gastrocopta mussoni* specimens identified by Pokryszko (1996) as they (1) have a high, long, lamellate parietoangular tooth (2) have a separated angular tooth (3) have a slightly rounded to angled columellar tooth and (4) lack an infraparietal tooth. They may prove to be a subspecies of *Gastrocopta larapinta* and in this sense, Pokryszkos’ (1996) separation of cylindrical *Gastrocopta mussoni* and *Gastrocopta larapinta* (no interpalatal tooth) was correct.

As there is doubt surrounding the distinguishing morphological characters of cylindrical *Gastrocopta mussoni* and *Gastrocopta larapinta* shells with a small or no interpalatal tooth, the above separation is tentative and a more detailed genetic investigation is required.

### 3. Gastrocopta margaretae

(Cox, 1868)

http://species-id.net/wiki/Gastrocopta_margaretae

Fig. 2F

Pupa margaretae Cox 1868: 80, pl. 14, figs 20,20a.
Pupa wallabyensis Smith 1894: 97.
Gastrocopta margaretae Pilsbry 1917 [in 1916–1918]: 160–161, pl. 26, figs 7–8; Solem 1986: 99–101, figs 8–9,11–12, 1991: 249; Pokryszko 1996: 1096–1099, figs 6, 8–10.
Gastrocopta tatei Pilsbry 1917 [in 1916–1918]: 165–166, pl. 26, figs 9–10, pl. 30, fig. 12; Solem 1989: 491–492, figs 56–59; 1991: 249.
Gastrocopta wallabyensis Pilsbry 1917 [in 1916–1918]: 171–172; Solem 1986: 101–102, figs 13–15; 1991: 249.
Australbinula margaretae Iredale 1937a: 302, 1937b: 11, pl. 1, fig. 4.
Australbinula wallabyensis Iredale 1937a: 302.
Australbinula tatei Iredale 1937a: 302, 1937b: 10.
Gastrocopta pilbarana Solem 1986: 103–104, figs 16–20; 1991: 249.

#### Type locality.

Wallaroo, South Australia.

#### Material studied.

Western Australia: Bateman Sanctuary: 23.0552°S, 113.8234°E (WAM S42834). ~18km N of Boolathana Homestead: 24.4133°S, 113.7445°E (WAM S64708). Boolathana Station: 24.4127°S, 113.7631°E (WAM S64709). Bush Bay: 25.1175°S, 113.8063°E (WAM S42806); 25.1316°S, 113.7681°E (WAM S60355); 25.1136°S, 113.7311°E (WAM S64575); 25.1175°S, 113.8063°E (WAM S64577). Carrarang Station: 26.1666°S, 113.3500°E (WAM S34378). Cy Creek: 23.1000°S, 113.8000°E (WAM S34380). Dirk Hartog Island: 25.7166°S, 113.0667°E (WAM S14439); 25.8333°S, 113.0500°E (WAM S34398). Francois Peron National Park: 25.9760°S, 113.5707°E (WAM S60269); 25.9758°S, 113.5706°E (WAM S64706); 25.8752°S, 113.5497°E (WAM S61127). Lake Macleod area: 24.3449°S, 113.5194°E (WAM S65084); 24.3668°S, 113.5145°E (WAM S65093); 24.3544°S, 113.5098°E (WAM S65102); 24.4760°S, 113.5257°E (WAM S65108); 24.4598°S, 113.5013°E (WAM S65110); 24.3544°S, 113.5098°E (WAM S65121). 0.25 miles W of Nichol Springs: 24.1333°S, 118.4167°E (WAM S60270). ~25 miles N of turn off to Shark Bay on NW Coastal Highway: 26.0647°S, 114.3353°E (WAM S34459, WAM S60271, WAM S64585). 0.5 miles W of 512 mile peg on NW Coastal Highway: 26.1966°S, 114.3758°E (WAM S34379). Quobba Station: 24.4758°S, 113.4166°E (WAM S42829); 24.2448°S, 113.5353°E (WAM S61125); 24.1927°S, 113.4548°E (WAM S64576); 24.2233°S, 113.5036°E (WAM S64707). Salutation Island: 26.5333°S, 113.7667°E (WAM S34377). Winderabandi Point: 22.4929°S, 113.7258°E (WAM S60474). Zuytdorp: 27.2636°S, 114.0703°E (WAM S64710).

#### Distribution.

This species has previously been recorded fromthe western and southern coastal areas of Western Australia, the southern regions of South Australia and the area near Alice Springs in the Northern Territory. There is also an isolated record from the King Leopold Range in the north of Western Australia (Pokryszko 1996). In the Pilbara it is confined to the near west coast with an isolated inland record from the Ashburton River (Figure 3).

#### Comparative morphology.

Shells of *Gastrocopta margaretae* are easily distinguished from other *Gastrocopta* species in the Pilbara by the presence of (1) a moderately to strongly folded columellar tooth (2) a generally large and transverse basal tooth (3) a high and long lower palatal tooth (4) an upper palatal tooth that is moderately to strongly convergent with the lower palatal (5) a weak to strong infraparietal tooth present.

#### Remarks.

Solem (1986) maintained the separation of the west coast species *Gastrocopta wallabyensis* from the south coast *Gastrocopta margaretae* based on size (smaller) and length of apertural barriers (longer). He also described a new species, *Gastrocopta pilbarana*, from the west coast but his separation of it from *Gastrocopta wallabyensis* was vague. Solem later (1989) maintained the separation of the central Australian *Gastrocopta tatei* from the above species but remarked it was somewhat similar to the west coast *Gastrocopta wallabyensis*. Pokryszko (1996) later disagreed, synonymising all species with *Gastrocopta margaretae*.

The few specimens sequenced from the south coast of Western Australia (WAM S32048, WAM S32052) could represent genetic isolation by distance or perhaps a different species from those on the west coast (WAM S42834) but more molecular data are required. The southern specimens are (1) much larger with reduced apertural barriers (2) more strongly rounded whorls (conical) and (3) consistently lack an infraparietal tooth. Specimens resembling the smaller west coast form (ie. long apertural barriers and weak to strong infraparietal tooth) have also been recorded from the south west area of Western Australia (Whisson, pers. comm.) where it is often sympatric with *Gastrocopta bannertonensis* (Gabriel, 1930). It is not known whether there is a continuous distribution between the two areas. Until more detailed molecular work is undertaken we have maintained Pokryszko’s (1996) systematic positions.

### 4. Gastrocopta mussoni

Pilsbry, 1917

http://species-id.net/wiki/Gastrocopta_mussoni

Fig. 2H–J

Gastrocopta larapinta deserti Pilsbry 1917 [in 1916–1918]: 170–171, pl. 30, figs 1–3.
Gastrocopta mussoni Pilsbry 1917 [in 1916–1918]: 167–168, pl. 27, figs 5–6; Solem 1989: 494, figs 211–213, 1991: 249; Pokryszko 1996: 1105–1109, figs 20–21; Stanisic et al. 2010: 102–103.
Australbinula helmsiana Iredale 1939: 8, pl. 1, fig. 2.
Australbinula mussoni Iredale 1937a: 301.
Gastrocopta deserti Solem 1986: 102–103, figs 13–15, 1989: 487–490, figs 48–53, 1991: 249; Slack-Smith 1993: 92.

#### Type locality.

Calliungal (=Mt Morgan), Queensland.

#### Material studied.

Western Australia: Angelo River: 23.4331°S, 118.7329°E (WAM S65935). Anketell Point area: 20.6356°S, 117.0398°E (WAM S59990); 20.6719°S, 117.0965°E (WAM S599912); 20.7025°S, 117.0473°E (WAM S80936). Area C: 22.9104°S, 118.9664°E (WAM S60417). Barrow Island: 20.7833°S, 115.4000°E°E (WAM S34384); 20.8649°S, 115.4069°E°E (WAM S34455); 20.6666°S, 115.4667°E (WAM S42879, WAM S60847); 20.7921°S, 115.4573°E (WAM S59636); 20.7858°S, 115.4573°E (WAM S59637); 20.7997°S, 115.4403°E (WAM S59640); 20.7069°S, 115.4194°E (WAM S59642); 20.7866°S, 115.4547°E (WAM S59644, WAM S60413); 20.7938°S, 115.4575°E (WAM S59651); 20.8644°S, 115.3428°E (WAM S59652); 20.8101°S, 115.4270°E (WAM S60410); 20.6666°S, 115.4667°E (WAM S42879, WAM S60847); 20.7977°S, 115.4064°E (WAM S65123, WAM S65126, WAM S65127, WAM S65164); 20.7684°S, 115.4673°E (WAM S65174). Cane River Conservation Park: 22.1694°S, 115.5606°E (WAM S42961); 22.4321°S, 115.2895°E (WAM S42967); 22.2685°S, 115.6470°E (WAM S42974); 22.0975°S, 115.4942°E (WAM S42976); 22.2075°S, 115.5260°E (WAM S42981); 22.1451°S, 115.7249°E (WAM S42987); 22.0298°S, 115.4296°E (WAM S42992); 22.0131°S, 115.6325°E (WAM S42998). Cape Preston area: 20.8435°S, 116.2016°E (WAM S59141). Chichester Ranges: 22.0525°S, 118.9883°E (WAM S60407); 22.0516°S, 118.9884°E (WAM S42711, WAM S42713, WAM S42759); 22.1508°S, 119.0179°E (WAM S42709); 22.0503°S, 118.9934°E (WAM S42717); 23.1164°S, 119.8865°E (WAM S64460). Christmas Creek area: 22.4170°S, 119.8941°E (WAM S65603); 22.4078°S, 119.8767°E (WAM S65605); 22.4061°S, 119.7376°E (WAM S65611). Cloud Break area: 20.3216°S, 119.4418°E (WAM S34460); 22.3210°S, 119.4442°E (WAM S42876, WAM S60403, WAM S80957); 22.3181°S, 119.3788°E (WAM S60267); 22.2935°S, 119.3872°E (WAM S61122); 22.2997°S, 119.3737°E (WAM S61123); 22.2881°S, 119.2360°E (WAM S65137°S, WAM S65143°S, WAM S65153); 22.3251°S, 119.4458°E (WAM S65139); 22.3527°S, 119.4189°E (WAM S65144); 22.3652°S, 119.3409°E (WAM S65148); 22.3894°S, 119.4380°E (WAM S65155); 22.3985°S, 119.4748°E (WAM S65157); 22.3949°S, 119.5019°E (WAM S65213). Cy Creek: 22.8183°S, 114.0609°E (WAM S34382). Dolphin Island: 20.4833°S, 116.8500°E (WAM S34385). Du Boulay Creek: 21.1833°S, 116.1833°E (WAM S34568). Finucane Island: 20.2982°S, 118.5572°E (WAM S16108). Fortescue Marsh area: 22.2822°S, 119.1276°E (WAM S42926, WAM S64694, WAM S646523); 22.2938°S, 119.0732°E (WAM S61991); 22.5098°S, 119.1274°E (WAM S61993°S, (WAM S61995); 22.2872°S, 119.0301°E (WAM S646401, WAM S646556); 22.1322°S, 119.1983°E (WAM S64684); 22.2924°S, 119.0279°E (WAM S64687). East Hamersley Range: 22.8586°S, 119.6728°E (WAM S42921); 22.6335°S, 119.3289°E (WAM S64470). Hope Downs: 23.1474°S, 119.5191°E (WAM S42662); 23.1030°S, 119.2917°E (WAM S59548, WAM S59554); 23.0925°S, 119.2058°E (WAM S59551); 23.0878°S, 119.1609°E (WAM S59552). Jinayri area: 22.9219°S, 119.2036°E (WAM S42929); 23.0530°S, 119.2701°E (WAM S59239, WAM S592412, WAM S59244); 23.0129°S, 119.2371°E (WAM S59240, WAM S59243); 22.9058°S, 119.2000°E (WAM S59576, WAM S59596); 22.9275°S, 119.1275°E (WAM S59585); 22.9169°S, 119.2128°E (WAM S59587); 22.9219°S, 119.2036°E (WAM S59591); 23.0504°S, 119.2731°E (WAM S60406). Kalgan Pool area: 23.1877°S, 119.6965°E (WAM S439823). Kangeenarina Gorge area: 22.1186°S, 117.9427°E (WAM S61763); 21.8489°S, 117.3837°E (WAM S81210). Karratha area: 20.7166°S, 116.8500°E (WAM S60412); 21.4036°S, 116.9392°E (WAM S81217). SE of Karratha: 21.0698°S, 116.9702°E (WAM S81241, WAM S81246); 21.0300°S, 116.9997°E (WAM S81252, WAM S81271); 20.9853°S, 116.8811°E (WAM S81268). Lake Macleod: 24.3544°S, 113.5098°E (WAM S65120). Marillana Station: 22.5840°S, 119.3114°E (WAM S84075); 22.5663°S, 119.2308°E (WAM S34473); 22.5497°S, 119.2147°E (WAM S34476); 22.5851°S, 119.3142°E (WAM S34478). 22.5739°S, 119.2562°E (WAM S34634, WAM S80940); 22.5538°S, 119.2283°E (WAM S34635); 22.6260°S, 119.2834°E (WAM S34639); 22.5625°S, 119.2311°E (WAM S80907); 22.1269°S, 119.2123°E (WAM S80910); 22.5625°S, 119.2311°E (WAM S80913); 22.4080°S, 119.0067°E (WAM S80916); 22.6376°S, 119.3744°E (WAM S80917); 22.4285°S, 119.2043°E (WAM S81437); 22.3182°S, 119.1175°E (WAM S81442); 22.4295°S, 119.1923°E (WAM S81444). Meentheena Outcamp: 21.2671°S, 120.4570°E (WAM S58055); 21.2815°S, 120.4511°E (WAM S58099); 21.2816°S, 120.4508°E (WAM S58098). Millstream National Park: 21.6000°S, 117.1000°E (WAM S42838, WAM S42931, WAM S61043); 21.5833°S, 117.0833°E (WAM S42839, WAM S60943); 21.5833°S, 117.0667°E (WAM S60946); 21.4255°S, 117.0535°E (WAM S81212); 21.1781°S, 117.0461°E (WAM S81250); 21.2039°S, 117.0440°E (WAM S81301, WAM S81296). Mount Brockman area: 22.4815°S, 117.2384°E (WAM S83561). Mt Farquhar area: 22.4815°S, 116.8108°E (WAM S83563). Muiron Island: 21.6666°S, 114.3333°E (WAM S34383). Murray Hills: 22.1147°S, 118.5221°E (WAM S59996). ~60km NW of Newman: 23.0530°S, 119.2701°E (WAM S60235, WAM S60266, WAM S60405); 23.0878°S, 119.1609°E (WAM S59549); 23.0504°S, 119.2731°E (WAM S60404, WAM S60411); 22.9632°S, 119.2276°E (WAM S42928, WAM S60233). ~112km NNE of Newman: 22.3621°S, 119.9691°E (WAM S65650); 22.3132°S, 119.8599°E (WAM S 65672); 22.3871°S, 119.9664°E (WAM S 65694). ~110km N of Newman: 22.2954°S, 119.8109°E (WAM S65638); 22.3132°S, 119.8599°E (WAM S65672); 22.2972°S, 119.8633°E (WAM S65683). ~70km S of Newman: 23.7270°S, 119.7242°E (WAM S58073). North Star: 21.2523°S, 118.8334°E (WAM S65699); 21.1971°S, 118.8286°E (WAM S65706, WAM S65710); 21.2681°S, 118.9682°E (WAM S65713); 21.2104°S, 118.8769°E (WAM S65718); 21.2104°S, 118.8769°E (WAM S65719); 21.2319°S, 118.8263°E (WAM S65728). Nullagine area: 21.8221°S, 120.3409°E (WAM S61802). Orebody 35°E (ca. 8km W of Newman): 23.4108°S, 119.5715°E (WAM S64733); 23.3837°S, 119.6478°E (WAM S64748); 23.3712°S, 119.6127°E (WAM S64749); 23.3819°S, 119.6133°E (WAM S64751); 23.3970°S, 119.6138°E (WAM S64752). West of Pannawonica: 21.7000°S, 116.1667°E (WAM S42805); 21.8063°S, 116.0774°E (WAM S60268); 21.7202°S, 116.0705°E (WAM S60414, WAM S61034); 21.6298°S, 116.0206°E (WAM S60415). Point Quobba, near lighthouse: 24.4797°S, 113.4178°E (FMNH 201611); Phils` Creek area: 22.7320°S, 119.1836°E (WAM S 42878, WAM S 59372); 22.7316°S, 119.1931°E (WAM S 59371, WAM S59375, WAM S59383); 22.7351°S, 119.1856°E (WAM S59373); 22.7384°S, 119.1916°E (WAM S59377); 22.7412°S, 119.1959°E (WAM S59378); 22.7448°S, 119.1927°E (WAM S59379); 22.7412°S, 119.1959°E (WAM S59380); 22.7366°S, 119.1811°E (WAM S59381); 22.7316°S, 119.1798°E (WAM S59382). ~40km S of Port Hedland: 20.6095°S, 118.6661°E (WAM S81404). NNE of Rocklea Homestead: 22.8101°S, 117.4734°E (WAM S80990). Roy Hill Station: 22.6642°S, 119.9458°E (WAM S34588); 22.4943°S, 119.9217°E (WAM S42828, WAM SS60376); 22.4396°S, 119.9453°E (WAM S42837); 22.4898°S, 119.8951°E (WAM S42875, WAM S60234); 22.5383°S, 119.9424°E (WAM S42927); 22.7058°S, 119.7082°E (WAM S60393); 22.6347°S, 119.9698°E (WAM S60394); 22.8174°S, 119.9473°E (WAM S60395, WAM S60399); 22.5771°S, 120.0247°E (WAM S60421, WAM S60866); 22.4793°S, 119.9421°E (WAM S60427); 22.5039°S, 120.0210°E (WAM S60864); 22.5566°S, 119.9700°E (WAM S60865); 22.7489°S, 119.9221°E (WAM S61067); 22.7195°S, 119.9395°E (WAM S61068, WAM S61071); 22.6365°S, 119.9639°E (WAM S61070); 22.6431°S, 119.9642°E (WAM S61074, WAM S61076); 22.5050°S, 119.9042°E (WAM S64448); 22.5843°S, 120.0172°E (WAM S64453).Running Waters (east of Nullagine): 21.6819°S, 121.1254°E (WAM S58050); 21.6815°S, 121.1270°E (WAM S58059). W end of Telfer Road: 21.3290°S, 121.1390°E (WAM S58044). NW of Tom Price: 22.3734°S, 117.4631°E (WAM S34566); 22.1350°S, 117.4728°E (WAM S65775); 22.1519°S, 117.5428°E (WAM S65781); 22.2997°S, 117.6378°E (WAM S65787); 22.3855°S, 117.4667°E (WAM S65788). Weeli Wolli Creek: 22.6166°S, 119.4000°E (WAM S60231). Near Wodgina Mine: 21.1831°S, 118.6569°E (WAM S34567, WAM S65843, WAM S65869); 21.2871°S, 118.8671°E (WAM S64618); 21.1789°S, 118.6463°E (WAM S65841, WAM S65855); 21.1737°S, 118.6503°E (WAM S65844); 21.2383°S, 118.6519°E (WAM S65903). Wonmunna: 23.1436°S, 119.0064°E (WAM S65977, WAM S81002, WAM S81026); 23.1355°S, 119.0384°E (WAM S65983); 23.1596°S, 118.9703°E (WAM S65979); 23.1632°S, 118.9770°E (WAM S65985); 23.1615°S, 119.0020°E (WAM S 65990); 23.1201°S, 119.0484°E (WAM S65993); 23.1287°S, 119.0904°E (WAM S 65996); 23.1283°S, 119.0736°E (WAM S65998); 23.1268°S, 119.0673°E (WAM S81032); 23.1309°S, 119.0689°E (WAM S81036, WAM S81167); 23.1428°S, 119.0099°E (WAM S81053); 23.1355°S, 119.0384°E (WAM S81081); 23.1169°S, 119.0396°E (WAM S81099); 23.1255°S, 119.0797°E (WAM S81094); 23.1216°S, 119.0498°E (WAM S81096, WAM S81171); 23.1393°S, 119.0182°E (WAM S81104); 23.1210°S, 119.0632°E (WAM S81109); 23.1356°S, 119.0463°E (WAM S 81110, WAM S81127); 23.1185°S, 119.0649°E (WAM S81114); 23.1255°S, 119.0797°E (WAM S81116); 23.1201°S, 119.0484°E (WAM S81119); 23.1331°S, 119.0154°E (WAM S81170); 23.1216°S, 119.0498°E (WAM S81178); 23.1220°S, 119.0611°E (WAM S81180). Yandi Mine: 22.8200°S, 119.2500°E (WAM S61774).

#### Distribution.

**Figure 5.**
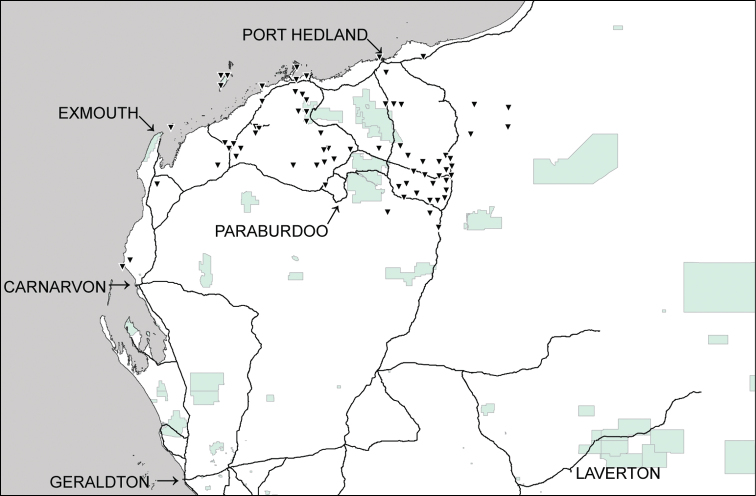
Distributional records of *Gastrocopta mussoni* Pilsbry, 1917 (▼) in the Pilbara region. Shaded: Protected area.

This species has previously been recorded from central Australia (the southern part of Northern Territory), with a few records from the mid-west coast and northern Western Australia; northern Northern Territory; northern and north-eastern parts of Queensland and South Australia (Pokryszko 1996). In addition, it is now recorded from throughout most of the Pilbara region (Figure 5).

#### Comparative morphology.

Cylindrical and elongate-ovate forms of *Gastrocopta mussoni* can be mistaken for *Gastrocopta larapinta* specimens (with a small or absent interpalatal tooth) but (1) are slightly to moderately slender (2) have a higher, usually longer parietoangular tooth (3) have a larger, more strongly slanted and acutely angled columellar tooth, with its posterior edge often forming a prominent wide ridge along the columellar wall (4) quite frequently have an upper palatal tooth that is slightly (occasionally moderately) convergent with the lower palatal (see also earlier section on *Gastrocopta larapinta*) . *Gastrocopta mussoni* very occasionally possesses a small interpalatal tooth, usually located close to the upper palatal.

The typical ovate form of *Gastrocopta mussoni* can be confused with *Gastrocopta hedleyi* (particularly those with reduced apertural barriers) but (1) are larger (obese) when sympatric (2) have a less sigmoid lower palatal tooth and (3) have a smaller, less convergent upper palatal tooth (see also earlier section on *Gastrocopta hedleyi*).

#### Remarks.

There appears to be two size forms in *Gastrocopta mussoni*, the larger ovate form and smaller, slender cylindrical form, and in agreement with Pokryszko (1996) both are confirmed as ecological phenotypes from the CO1 and 16S sequences. Based on specimens identified by Pokryszko (1996) and during this study, there is enormous variation in shell shape, shell size and apertural barrier structure between and including these two forms.

The ovate form of *Gastrocopta mussoni* appears to be most common in the Pilbara. Prior to Pokryszko’s 1996 publication, *Gastrocopta pilbarana* Solem, 1986 was described from the Shark Bay area with an isolated record from the Chichester Range (north of Roy Hill). This species was synonymised with *Gastrocopta margaretae* (Cox, 1868) by Pokryszko (1996) although the Chichester Range paratype was not included in that study. This Chichester Range record is actually the ovate form of *Gastrocopta mussoni*.

Some of those specimens tentatively identified as the elongate-ovate form of *Gastrocoptamussoni* during this study (Wonmunna; Cy Creek; Cloud Break, Barrow Island) are (1) much larger (obese) than the usual elongate-ovate form (2) have the parietoanangular tooth lower (45^o^) (3) usually have a supraparietal tooth and (4) quite frequently possess a small interpalatal tooth. These specimens may prove to be the somewhat variable *Gastrocoptalarapinta* with a small or no interpalatal tooth, but in the absence of a larger series of specimens and more detailed molecular data, we have left them as *Gastrocopta mussoni*.

The nature of many cylindrical *Gastrocopta mussoni* identified by Poykrosko (1996) and during this study requires more work. It is probable we have lumped the slender form of *Gastrocopta larapinta* from Kalgan Pool *(*no interpalatal tooth) withcylindrical *Gastrocopta mussoni*.

### 5. Gastrocopta sp. CW1

Fig. 2A

Gastrocopta pilbarana Slack-Smith 1993: 91.

#### Material studied.

Western Australia: Barrow Island: 20.7069°S, 115.4194°E (WAM S59641). Cape Range No. 2 Deep Well: 21.9500°S, 114.0333°E (WAM S14132). Cape Range (cave): 22.1166°S, 113.9833°E (WAM S34394); 22.1500°S, 114.0000°E (WAM S34395, WAM S60409, WAM S60831); 22.0833°S, 113.9833°E (WAM S34396); 22.1833°S, 113.9833°E (WAM S80955). Exmouth rubbish tip: 21.9166°S, 114.1167°E (WAM S60408)

#### Distribution.

This species is recorded from the Cape Range and from an isolated site on Barrow Island (Figure 3).

#### Comparative morphology.

Shells of *Gastrocopta* sp. CW1 are easily recognized by their (1) small size (2) very solid, non-lamellate columellar tooth that projects horizontally from the columellar wall (shelf-like), slightly drooping at anterior end (3) long sigmoid lower palatal tooth (4) large, transverse upper palatal tooth (5) presence of a suprapalatal tooth.

#### Remarks.

Solem (1989) identified specimens from the Kimberley and Northern Territory as *Gastrocopta recondita* (Tapparone-Canefri, 1883) but in a later review, Pokryszko (1996) regarded that species as extralimital to Australia, describing the Australian representatives as a new sister species, *Gastrocopta stupefasciens*.

*Gastrocopta* sp. CW1. is very similar to *Gastrocopta stupefaciens* and *Gastrocopta recondita* but (1) is smaller (2) has longer apertural barriers and (3) has a thick, solid, non-lamellate columellar tooth and is here within regarded as a new species. Some of Solems` *Gastrocopta recondita* specimens from limestone outcrops near Katherine (station WA-685) and Lake Argyle (station WA-248) have a similar columellar tooth structure and their relationship to *Gastrocopta* sp. CW1 needs further work.

Slack-Smith (1993) listed cavernicolus specimens from the Cape Range as *Gastrocopta pilbarana* (which was later synonymised with *Gastrocopta margaretae*) but those specimens were in fact *Gastrocopta* sp. CW1. She suggested that although this population of snails was ameliorated with the limestone caves of the Cape Range, although it was not generally cavernicolus. The accumulation and breakdown of leaf litter within caves combined with calcareous rocks was deemed advantageous for snails. Solem (1991) discussed an affinity with limestone for his *Gastrocopta recondita*. The few records of *Gastrocopta* sp. CW1 from the limestone dominated Barrow Island and Cape Range show similar requirements.

### 6. Gastrocopta servilis

(Gould, 1843)

http://species-id.net/wiki/Gastrocopta_servilis

Fig. 2G

Pupa servilis Gould 1843: 356, pl. 6, fig. 14.
Pupa microsoma Tapparone-Canefri 1883: 107–8, pl. 2, figs 1–2.
Pupa lyonsiana Ancey 1892: *Fr*. 5,713.
Gastrocopta lyonsiana Pilsbry 1917: 141–144, pl. 24, figs 1–4; van Benthem Jutting 1952: 355, fig. 34.
Gastrocopta microsoma (Tapparone-Canefri), Pilsbry 1917: 152, pl. 24, fig. 9; van Benthem Jutting 1964: 4–5
Gastrocopta servilis Solem 1989: 483–4, figs 38–41, 1991: 249; Shea 2006: 7, fig. 4; Stanisic et al. 2010: 104, fig. 123.

#### Type locality.

near Matanzas, Cuba.

#### Material studied.

Karratha area: 20.7385°S, 116.8357°E (WAM S9932, WAM S60237).

#### Distribution.

This species has previously been recorded from just north of Broome (Quondong Point) across northern Australia to mid-eastern Queensland and offshore islands (Solem 1989, Shea 2006). In addition, it is now recorded from a single locality within the Karratha town site (Figure 4).

#### Comparative morphology.

The shells of *Gastrocopta servilis* are easily distinguished from other Pilbara *Gastrocopta* by their (1) strongly rounded whorls (2) short, straight columellar tooth which is perpendicular to the mid-columellar wall (3) very long angular tooth which is fused with the parietoangular tooth (4) weak to absent basal tooth and (5) weak to absent upper palatal tooth.

#### Remarks.

*Gastrocopta servilis* has been a recent introduction to the residential gardens of Karratha.

## Molecular phylogeny

**Figure 6.**
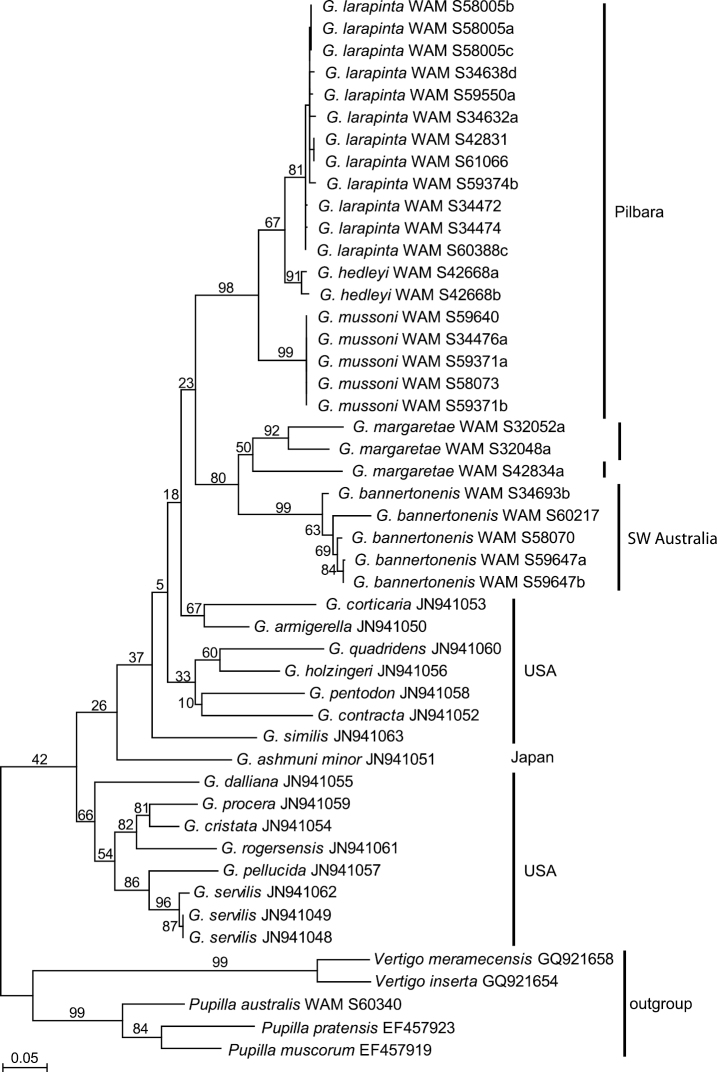
Maximum Likelihood phylogram for COI based on analysis of 27 new sequences of *Gastrocopta* from Western Australia and the 16 Genbank sequences made available by Nekola et al. (2012). Sequences of *Vertigo* spp. and *Pupilla* spp. were used as out-group to root the tree. Labels on branches indicate nodal support by 200 ML bootstrap replicates.

**Figure 7.**
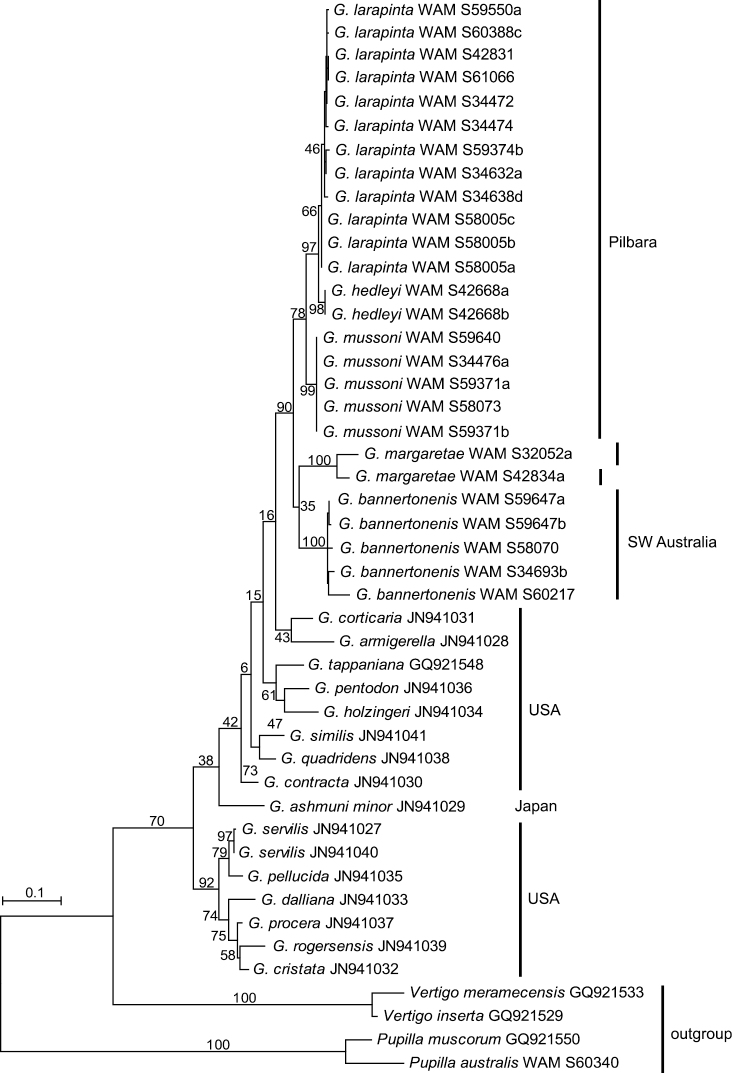
Maximum Likelihood phylogram for 16S based on analysis of 26 new sequences of *Gastrocopta* from Western Australia and the 16 Genbank sequences made available by Nekola et al. (2012). Sequences of *Vertigo* spp. and *Pupilla* spp. were used as out-group to root the tree. Labels on branches indicate nodal support by 200 ML bootstrap replicates.

Two mitochondrial gene fragments, COI and 16S, have been analysed. The data sets contained 27 sequences of Western Australian *Gastrocopta* (five each of *Gastrocopta bannertonensis* and *Gastrocopta mussoni*, 12 of *Gastrocopta larapinta*, two or three, respectively, of *Gastrocopta margaretae*, and two of *Gastrocopta hedleyi*) as well as 16 Genbank sequences of several American *Gastrocopta* species that stem from the study of Nekola et al. (2012). Two to three sequences each of *Vertigo* spp. and *Pupilla* spp. were used as out-group to root the trees. Maximum Likelihood analyses of the COI and 16S fragments resulted in identical tree topologies (Figs 6–7). All species as delineated by their shell formed monophyletic sequence clusters. The six Australian species formed a monophyletic crown group nested amongst a basal assemblage of American lineages. The species *Gastrocopta hedleyi*, *Gastrocopta mussoni* and *Gastrocopta larapinta* are more closely related with each other as are *Gastrocopta margaretae* and *Gastrocopta bannertonensis*, which corresponds well with columellar tooth structure i.e. large and ascending versus small and short, respectively. Intraspecific evolutionary divergences were on average 1% (max. 4%) in COI as well as on average 1% (max. 2%) in 16S in all Australian species but *Gastrocopta margaretae* (Tables 2–3). In *Gastrocopta margaretae* intraspecific genetic distances were found to be significantly higher than in any other Australian species (16% in COI and 5.3% in 16S). Apart from *Gastrocopta margaretae*, the intraspecific divergence was about an order of magnitude smaller than the observed interspecific distances of 5–26% (on average 18%) in COI and 2–14% (on average 9%) in 16S. Only in *Gastrocopta margaretae* did the amount of intraspecific genetic differentiation overlap with the range of interspecific genetic distances.

**Table 2.** Average pair-wise genetic distances in COI. Shown are (p) uncorrected p-distances, and (TN93) corrected distances by using the model of Tamura-Nei (1993). The rate variation among sites was modelled with a gamma distribution (shape parameter = 0.4).

	**Distance**	***G. marg***	***G. bann***	***G. lara***	***G. muss***	***G. held***
*G. marg*	p	0.103	0.121	0.133	0.128	0.129
	TN93	0.160	0.195	0.218	0.202	0.204
*G. bann*	p		0.030	0.147	0.132	0.142
	TN93		0.036	0.263	0.211	0.244
*G. lara*	p			0.008	0.080	0.044
	TN93			0.008	0.113	0.052
*G. muss*	p				0.000	0.081
	TN93				0.000	0.115
*G. held*	p					0.012
	TN93					0.013

**Table 3.** Average pair-wise genetic distances in 16S. Shown are (p) uncorrected p-distances, and (TN93) corrected distances by using the model of Tamura-Nei (1993). The rate variation among sites was modelled with a gamma distribution (shape parameter = 0.4).

	**Distance**	***G. marg***	***G. bann***	***G. lara***	***G. muss***	***G. held***
*G. marg*	p	0.045	0.102	0.094	0.082	0.095
	TN93	0.053	0.139	0.126	0.103	0.126
*G. bann*	p		0.021	0.080	0.076	0.076
	TN93		0.024	0.106	0.096	0.099
*G. lara*	p			0.008	0.040	0.021
	TN93			0.008	0.046	0.023
*G. muss*	p				0.000	0.041
	TN93				0.000	0.046
*G. held*	p					0.000
	TN93					0.000

## Discussion

Based on shell morphology, all *Gastrocopta* species recorded here (except *Gastrocopta* sp. CW1) have relatively large distributional ranges. Although limited, the molecular data supports the shell-based delineation of the six species recognized herein. The molecular data also confirms the large distributional ranges of *Gastrocopta larapinta* and *Gastrocopta mussoni* by including samples from areas that are about 100 and 550 kilometres apart, respectively. The apparently widespread distribution of *Gastrocopta* species is probably due to the common ability in which a single *Gastrocopta* adult can self-fertilize their eggs and establish a new population (Nekola 2009). They also have the ability to be transported large distances, through either their small and light structure (via wind and water) and/or by their nature to mucous seal to objects such as bark, leaves and vertebrates (Slack-Smith 1993, Nekola 2009).

Some of the species *Gastrocopta hedleyi* and *Gastrocopta* sp. CW1 are at the southern limits of their range. It is probable that *Gastrocopta hedleyi* has arrived in the Pilbara as a result of dispersal potential whereas *Gastrocopta* sp. CW1, found only in the Cape Range in the Pilbara (and an isolated record from Barrow Island) might represent relictual populations from the Miocene. Both Cape Range and Barrow Island contain moist, well sheltered limestone gorges and caves.

Other recorded species represent a range extension from the red centre. These include *Gastrocopta mussoni* and *Gastrocopta larapinta* which are common in the Pilbara. This is not suprising given their affinity to arid or semi-arid environments, which persist throughout much of the Pilbara. Future collecting will no doubt show a mostly continuous distribution for these species between the Pilbara and the red centre.

The present CO1 and 16S molecular data set, although small (only 27 individuals sequenced) mostly supports the taxonomic revision of Pokryszko (1996) based exclusively on shell morphology (i.e. apertural barriers). However, more detailed molecular work is needed to sort out some systematic issues: (1) the relationship of the west coast and south coast populations of *Gastrocopta margaretae* (2) the relationship of *Gastrocopta* sp. CW1 to similar specimens in the Kimberley region (3) the morphological separation of *Gastrocopta larapinta* and *Gastrocopta mussoni*.

The Australian species are less well differentiated by means of evolutionary divergence than the American *Gastrocopta* species, which are separated from each other by interspecific pair-wise Tamura and Nei (1993) distances of 7.8–28% (on average 20.4%) in COI and 2.3–22% (on average 13.9%) in 16S. Evolutionary divergences of the Australian *Gastrocopta* species are also lower than the genetic distances found in other Western Australian land snails, such as the Camaenidae. In this group interspecific sequences in COI were usually larger than 6% (e.g., Köhler 2011; Köhler and Johnson 2012) (16S distances were not compared because the analysed gene fragments differed in length).

There appears to be tremendous variation in shell shape and size between and within populations of some *Gastrocopta* species, and often this is associated with variation in apertural barrier structure. This can make separation of species difficult, particularly *Gastrocopta larapinta*, *Gastrocopta hedleyi* and *Gastrocopta mussoni* which share similar apertural barrier structures. It is advisable to collect a large series of individuals so the wide variation in apertural barrier structures can be seen.

## Conclusion

In summary, *Gastrocopta hedleyi*, *Gastrocopta larapinta*, *Gastrocopta*. sp. *CW1* and *Gastrocopta servilis* are recorded from the Pilbara region for the first time. *Gastrocopta servilis* has been a recent introduction to the residential gardens of Karratha. *Gastrocopta hedleyi*, *Gastrocopta larapinta* and *Gastrocopta mussoni* were shown to be common across the Pilbara. *Gastrocopta* sp. CW1may represent an undescribed species.

## Supplementary Material

XML Treatment for Gastrocopta hedleyi

XML Treatment for Gastrocopta larapinta

XML Treatment for Gastrocopta margaretae

XML Treatment for Gastrocopta mussoni

XML Treatment for Gastrocopta sp. CW1

XML Treatment for Gastrocopta servilis
